# FDA-Approved Inhibitors of RTK/Raf Signaling Potently Impair Multiple Steps of In Vitro and Ex Vivo Influenza A Virus Infections

**DOI:** 10.3390/v14092058

**Published:** 2022-09-16

**Authors:** Robert Meineke, Sonja Stelz, Maximilian Busch, Christopher Werlein, Mark Kühnel, Danny Jonigk, Guus F. Rimmelzwaan, Husni Elbahesh

**Affiliations:** 1 Research Center for Emerging Infections and Zoonoses (RIZ), University of Veterinary Medicine in Hannover (TiHo), Bünteweg 17, 30559 Hannover, Germany; 2 Institute of Pathology, Hannover Medical School (MHH), Carl-Neuberg-Straße 1, 30625 Hannover, Germany; 3 Member of the German Center for Lung Research (DZL), Biomedical Research in Endstage and Obstructive Lung Disease Hannover (BREATH), Hannover Medical School (MHH), Carl-Neuberg-Straße 1, 30625 Hannover, Germany

**Keywords:** influenza, antivirals, kinases, innate immunity, respiratory viruses, inhibitors

## Abstract

Influenza virus (IV) infections pose a burden on global public health with significant morbidity and mortality. The limited range of currently licensed IV antiviral drugs is susceptible to the rapid rise of resistant viruses. In contrast, FDA-approved kinase inhibitors can be repurposed as fast-tracked host-targeted antivirals with a higher barrier of resistance. Extending our recent studies, we screened 21 FDA-approved small-molecule kinase inhibitors (SMKIs) and identified seven candidates as potent inhibitors of pandemic and seasonal IV infections. These SMKIs were further validated in a biologically and clinically relevant ex vivo model of human precision-cut lung slices. We identified steps of the virus infection cycle affected by these inhibitors (entry, replication, egress) and found that most SMKIs affected both entry and egress. Based on defined and overlapping targets of these inhibitors, the candidate SMKIs target receptor tyrosine kinase (RTK)-mediated activation of Raf/MEK/ERK pathways to limit influenza A virus infection. Our data and the established safety profiles of these SMKIs support further clinical investigations and repurposing of these SMKIs as host-targeted influenza therapeutics.

## 1. Introduction

Influenza viruses (IVs) are important human respiratory pathogens that cause yearly epidemics and sporadic pandemics, resulting in substantial global morbidity and mortality [1]. At-risk populations including children, the elderly and the immunocompromised are at an especially higher risk of developing severe disease; yearly influenza vaccination is especially recommended for these populations [2,3,4,5,6]. Due to the emergence of antigenic drift variants, influenza vaccines are often updated annually to antigenically match the currently circulating strains [7,8]. However, effective vaccines are not readily available during influenza pandemics caused by novel and antigenically distinct IVs; this, coupled with the limited range of available IV antivirals to which resistance variants have emerged, highlights a need for additional therapeutic options. Currently licensed IV antivirals are direct-acting antivirals (DAAs) that target viral components. Adamantanes targeting the M2 ion channel have been rendered ineffective due to the circulation of resistant viruses and are no longer in use [9]. However, licensed DAAs still in clinical use include neuraminidase inhibitors (NAIs) and recently developed polymerase inhibitors such as baloxavir, favipiravir and pimodivir, all of which target individual viral proteins of the polymerase complex. NAIs, such as oseltamivir, have resulted in the emergence of resistant strains (4–5% in adult and 5–18% in pediatric patients) [10,11]. Similarly, a high frequency (~10%) of baloxavir-resistant viruses have been detected in healthy adults and adolescents, and the frequency is even higher in immunocompromised patients [12,13]. Thus, there are viruses currently circulating in human populations that are resistant to at least one of the approved antivirals, highlighting the need for alternatives to DAAs.

Host-directed antivirals (HDAs) target host factors that are critical for virus replication and have become an attractive alternate approach to DAAs. HDAs are less susceptible to escape mutations associated with DAAs, and a significant number of viral mutations would be required to use alternative or redundant pathways, typically resulting in a significant loss of viral fitness [14]. Host kinases regulate viral entry, RNA replication, viral release and innate immune signaling [15,16,17,18,19,20]. Moreover, viral protein phosphorylation is often critical for viral replication and evasion/suppression of innate immune responses. Several signaling pathways have been reported to play a critical role in influenza A virus (IAV) infections. Inhibition of either the receptor (RTKs) or non-receptor (NRTKs) tyrosine kinases in these pathways has been shown to have a deleterious effect on IAV infections [15,18,21]. 

We previously demonstrated the antiviral activity of FDA-approved NRTK inhibitors (NRTKIs) against IAV [22]. Our present study builds on those data by investigating the efficacy of 21 additional FDA-approved small-molecule kinase inhibitors (SMKIs), mostly targeting RTKs, NRTKs and serine/threonine kinases, against in vitro IAV infections. We validated the efficacy of our most promising inhibitors and further tested for their antiviral activity in human precision-cut lung slices (hPCLSs). Because hPCLSs allow cell–cell interactions that are crucial for viral tissue tropism, infectivity and host responses, they represent an important biologically relevant ex vivo model for IAV infections [23,24,25,26]. Finally, we elucidated the steps of the virus replication cycle that were inhibited by the respective SMKIs. Collectively, these inhibitors hold promise as HDAs against influenza virus infections and warrant further clinical investigation. 

## 2. Materials and Methods

### 2.1. Cells and Viruses

Madin–Darby canine kidney (MDCK) cells were grown in Dulbecco’s modified Eagle medium (DMEM) supplemented with 10% fetal bovine serum (FBS), 100 IU/mL penicillin, 100 µg/mL streptomycin, 2 mM glutamine and 1% non-essential amino acids (NEAAs). Human lung carcinoma (A549) cells were grown in Ham’s F-12 K-Nut Nutrient Mix medium supplemented with 10% FBS, 100 IU/mL penicillin, 100 µg/mL streptomycin and 2 mM GlutaMAX. Cells were cultured at 37 °C with 5% CO_2_. All culture media and supplements were purchased from Thermo Fisher Scientific (Asheville, NC, USA). 

Pandemic H1N1 influenza virus strain A/Netherlands/602/09 (NL09) and seasonal H3N2 influenza virus strain A/Netherlands/241/11 (NL11) were sourced from the Repository of the National Influenza Center at the Erasmus Medical Center in Rotterdam, the Netherlands, and were grown on MDCKs for 48 h at 37 °C. Virus stocks and culture supernatants were stored at −80°C until further use. Virus samples were titrated in MDCK cells by a median tissue culture infectious dose (TCID_50_) assay using the method of Reed and Muench [27].

### 2.2. Human Precision-Cut Lung Slices (hPCLSs) 

hPCLSs for ex vivo research were generated using lung tissues acquired from patients undergoing surgery at the Hannover Medical School. An expert pathologist verified that the tissues utilized were tumor-free after they were acquired from lung tumor resections. As previously mentioned, newly acquired lung tissues were processed into circular slices 300 microns thick and 8 mm in diameter [24]. All donors gave informed consent, as authorized by the Ethics Committee of Hannover Medical School (Ethics vote #8867 BO K 2020). PCLS were cultured in DMEM/F12 media (ThermoFisher Scientific, Asheville, NC, USA) supplemented with 2 mM HEPES, 1 mM GlutaMAX, 100 U/mL penicillin and 100 g/mL streptomycin in a humidified 37 °C/5% CO_2_ incubator. The hPCLSs were cultured for 4–8 weeks with no notable changes in cell type or morphology; cilia beating could be seen in all hPCLSs. 

### 2.3. Inhibitors 

All small-molecule kinase inhibitors (SMKIs) were purchased from Selleckchem (Houston, TX, USA). Inhibitors were diluted in DMSO to 10 mM stocks and stored at −20 °C until use.

### 2.4. In Vitro and Ex Vivo Cytotoxicity Assays 

The in vitro cytotoxicity of SMKIs was assessed using the CellTiter-Glo 2.0 (CTG) Cell Viability Assay (Promega, Madison, WI, USA) according to the manufacturer’s protocol. The cytotoxicity of SMKIs was assessed using the LDH-Glo Cytotoxicity Assay (Promega, Madison, WI, USA) according to the manufacturer’s protocol on mock- and/or virus-infected hPCLSs. The supernatants of SMKI-treated and untreated hPCLSs were collected and replaced entirely with new pre-warmed infection medium containing the relevant concentrations of SMKIs. LDH levels were normalized to a positive control (treated for 30 min at 37 °C with 1% Triton-X 100).

### 2.5. Virus Infections

For in vitro infections, semi-confluent (80–90%) A549 cells were infected at the indicated MOI with NL09 or NL11 diluted in F12K medium supplemented with 0.1% bovine serum albumin (BSA) and 50 ng/mL TPCK-treated trypsin at 37 °C. For ex vivo infections, hPCLSs were infected with NL09 or NL11 at 10^5^ TCID_50_/200 uL diluted in DMEM/F12 media supplemented with 2 mM HEPES, 1 mM GlutaMAX, 100 U/mL penicillin, 100 g/mL streptomycin and 200 ng/mL TPCK-treated trypsin at 37 °C. After 1 h, the cells were washed twice with phosphate-buffered saline containing Mg^2+^/Ca^2+^ (PBS+/+) to remove unbound virus and then incubated at 37 °C in infection medium +/− the indicated SMKIs. Supernatants were collected and viral titers were assessed by a TCID_50_ assay in MDCK cells [27]. The assay’s lower limit of detection (LoD) is 10^1^ TCID_50_/_mL_, and its upper LoD is 10^9.5^ TCID_50_/_mL_.

### 2.6. Immunofluorescent Staining and Imaging

Cells were fixed with 4% paraformaldehyde (4% PFA) for 30 min at room temperature (RT), permeabilized with 0.1 percent Triton X-100 for 15 min at RT, washed with PBS and blocked for 1 h with heat-inactivated 5% horse serum in PBS (PBS-HS). Cells were then incubated overnight at 4 °C with mouse monoclonal antibodies to IAV nucleoprotein (clone HB65, American Type Culture Collection, Manassas, VA, USA) diluted in PBS-HS at 0.2 µg/mL. Cells were washed and incubated for 1 h at room temperature with AlexaFluor-594-conjugated goat anti-mouse IgG antibody (0.2 µg/mL) and NucBlue Live ReadyProbes Reagent (ThermoFisher Scientific, Asheville, NC, USA). Images were collected using a Leica DMi8 fluorescent microscope (Leica, Wetzlar, Germany), and quantitative analysis was conducted using the ImageJ (NIH, Bethesda, MD, USA) Threshold, Watershed and Particle Analyzer tools, adapted from [28] (*n* = 4). The total number of cells was determined by the nucleus count per 0.6 mm^2^. The number of infected cells per 0.6 mm^2^ was determined using NP staining. The infected-to-total cell ratio was used to calculate relative infectivity. Relative viability was defined as the ratio of the number of treated infected to treated uninfected cells for each SMKI. Prism 9 Heatmap (GraphPad, San Diego, CA, USA) function was used for visualization.

### 2.7. Polymerase Activity Assay

Lipofectamine LTX was used to transfect semi-confluent (70–80%) A549 cells (8 × 10^4^ cells in 24-well plates) with the pPOLI-358-FFLuc reporter plasmid, which expresses a firefly luciferase gene under the control of the viral nucleoprotein (NP) promoter (kindly provided by Megan Shaw) [29,30,31]. As a transfection control, the pmaxGFPTM expression (Lonza, Basel, Switzerland) vector was co-transfected.

**Figure 1:**
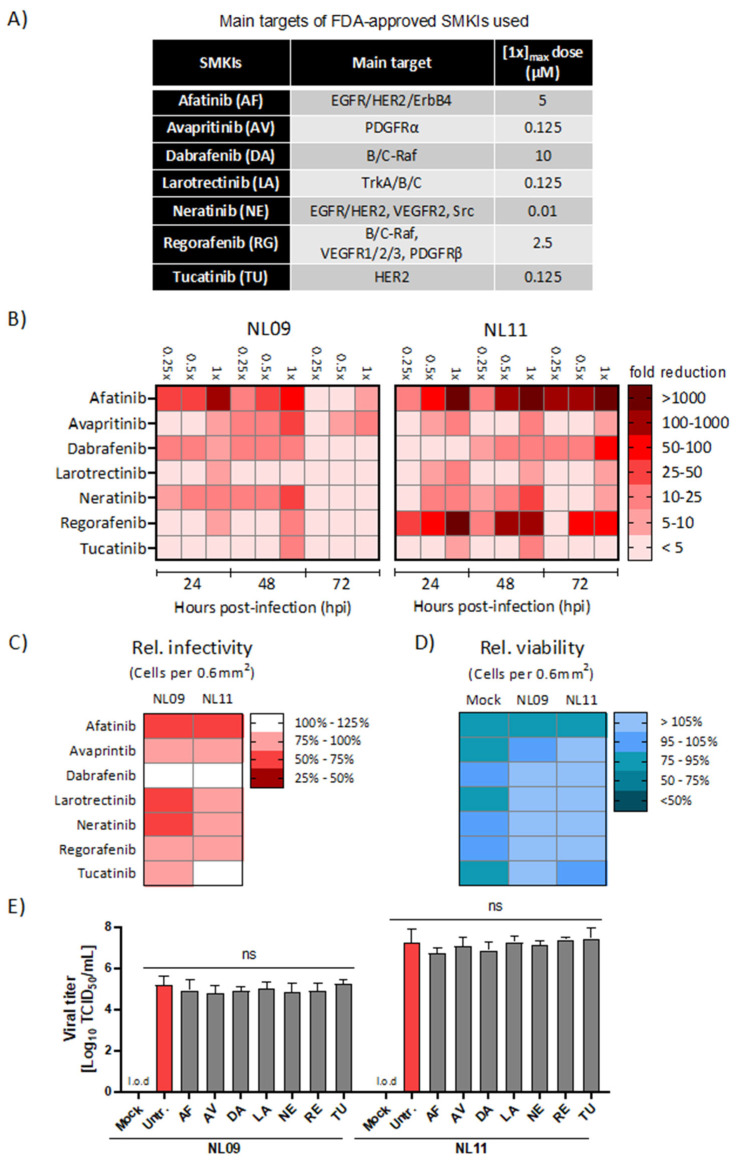
Effect of SMKI treatment on IAV replication, infectivity and viability. (**A**) Main targets and applied dosages of FDA-approved SMKIs used in this study. (**B**) A549 cells were infected with NL09 or NL11 at MOI = 1 +/− the indicated SMKIs at [0.25×, 0.5× or 1×]_max_ concentration up to 72 hpi. Viral titers were quantified by a TCID_50_/mL assay at 24, 48 and 72 hpi and visualized using a heatmap of the fold-change in viral titers relative to DMSO treatment (*n* = 4/condition). Additionally, see Figure S2. (**C**,**D**) A549 cells were infected with NL09 or NL11 at MOI = 1 and incubated for 48 h in the presence of SMKIs ([0.5×]_max_ concentration). Fluorescence microscopy images were acquired from cells stained for infected cells by anti-IAV NP antibody (red), and nuclei by using NucBlue Live ReadyProbes (blue). Data visualized in the heatmap are % infectivity (**C**) and % cell viability (**D**) relative to untreated infected cells or mock-infected treated cells (*n* = 4/condition). Additionally, see Figure S3. Images were quantified using ImageJ software. (**E**) NL09 and NL11 virus stocks were preincubated with the control (DMSO) or the [1×]_max_ concentration of the respective SMKI for 4 h at 37 °C. A549 cells were then infected using a 1:1000 dilution. Viral titers were determined at 72 h by a TCID_50_/mL assay (*n* = 3). Means ± SDs are shown. l.o.d.: limit of detection. ns, not significant. *p*-values were determined by Welch t-tests compared to untreated cells.

To determine minigenome polymerase activity, a mixture of plasmids containing the PB2, PB1, PA and NP genes from NL09 or A/NL/213/03 (H3N2) IAVs was co-transfected with the reporter and control plasmids in amounts of 0.35, 0.35, 0.35 and 0.5 µg. At 6 h post-transfection (hpt), the specified RTKIs were administered at 1× and 0.5× doses (see Figure 1A), and luciferase reporter activity was measured using the One-Glo luciferase assay kit (Promega, Madison, WI, USA) at 30 hpt (24 h of treatment). The mean fluorescence intensity (MFI) of GFP was determined, as well as the luciferase luminescence, using a F200 Pro multi-mode plate reader (Tecan, Männedorf, Switzerland).

To determine polymerase activity during IAV infection, cells were infected at an MOI of 1 with NL09 or NL11 24 hpt with the pPOLI-358-FFLuc reporter and GFP plasmids +/− the indicated SMKIs. At 48 hpt (24 hpi), the One-Glo luciferase assay (Promega, Madison, WI, USA) was used to measure luciferase reporter activity. The mean fluorescence intensity (MFI) of GFP was determined, as well as the luciferase luminescence, using a F200 Pro multi-mode plate reader.

### 2.8. Viral Entry Assay and Confocal Microscopy

A549 cells were seeded in 24-well plates on 12.5 mm coverslips. On the day of infection, cells were rinsed 3 times with PBS+/+ and incubated for 2 h in infection medium +/− the indicated SMKIs. The cells were cooled on ice for 15 min before inoculation with the virus (MOI = 10) at 4 °C on ice for 30 min +/− the indicated SMKIs. To minimize receptor activation caused by persistent viral-receptor engagement/internalization after 4 °C adsorption and to gently warm up the cells, unbound/noninternalized virus was removed by washing the cells twice with RT PBS+/+. The cells were subsequently incubated at 37 °C for 30 min with a pre-warmed infection containing the respective RTKIs. Cells were then fixed in 4% PFA, permeabilized for 15 min at room temperature with 0.1% Triton X-100, washed in PBS and incubated overnight at 4 °C in blocking buffer (PBS-HS). The cells were then incubated for 1 h at room temperature with anti-IAV NP antibody (clone HB65), rinsed three times with PBS and then incubated for 1 h at room temperature with AlexaFluor488-conjugated donkey anti-mouse IgG secondary antibody (0.2 µg/mL) diluted in PBS-HS. NucBlue Live ReadyProbes and ActinRed-555 ReadyProbes Reagent were used to stain cell nuclei and F-Actin, respectively. Prolong mounting medium (ThermoFisher Scientific, Asheville, NC, USA) was used to attach coverslips, and cell images were collected using a Leica TSC SP5 laser scanning confocal system mounted on an upright Leica DM6000 CFS and equipped with a 63× oil immersion objective. The photos were combined and analyzed using Leica LAS software (LASx ver. 3.7.2.22838, Leica, Wetzlar, Germany) across all experiments using identical camera settings.

### 2.9. SMKI Resistance Analysis

Semi-confluent MDCK cells (~10^6^ cells/well in 6-well plates) were infected with pandemic NL09 or seasonal NL11 at MOI = 0.001 at 37 °C for 72 h, +/− the SMKIs at [1×]_max_. As a control, viruses were also passaged in parallel without SMKIs. At each passage, viral titers were determined by a TCID_50_/_mL_ assay and used to infect cells in the subsequent passage at MOI = 0.001. Viruses were passaged 5 times.

### 2.10. Viral Egress Assay and qPCR Analysis

A549 cells were cooled on ice for 15 min before being inoculated with NL09 (MOI = 5) for 30 min on ice. Virus particles that were neither bound nor internalized were removed by washing the cells twice with RT PBS+/+. After that, the cells were cultured for 24 h at 37 °C/5% CO_2_ in pre-warmed infection medium without TPCK-treated trypsin and [1×]_max_ SMKI concentrations. At 24 hpi, the supernatant was collected, and cells were washed twice with PBS+/+ to remove any external virus. The supernatant and cells were used to isolate intra- and extracellular RNA, respectively, using the QIAamp Viral RNA Mini Kit (QIAGEN, Hilden, Germany). During the RNA isolation, 10 uL supernatant from canine distemper virus (CDV)-infected cells was supplied to each sample as an internal control. Complementary DNA (cDNA) was generated from the RNA isolates using the RevertAid First Strand cDNA Synthesis Kit (ThermoFisher Scientific, Ashveville, NC, USA) and the supplied random hexamer primer. We used 1 ug of cDNA template in each reaction to run a taqman multiplex qPCR using the Luna Universal Probe qPCR Master Mix (New England Biolabs, Ipswich, MA, USA). We used primers and probes specific for the A(H1)pdm09 HA gene, CDV and hGAPDH (Thermo Scientific Scientific, Asheville, NC, USA). HA primer and probe sequences were obtained from the WHO guidelines for the molecular detection of influenza viruses [32]. All qPCR reactions were performed in a LightCycler 480 (Roche, Basel, Switzerland) according to the reaction conditions indicated in [27,32]. All Ct values obtained were adjusted to their CDV and GAPDH Ct values. The relative ratio of intracellular Ct to extracellular Ct (I/E) was calculated using normalized HA Ct values. We calculated the ratio for each RTKI treatment condition relative to untreated infected cells.

### 2.11. Statistical Analyses

Statistical analyses in Prism 9 (GraphPad, San Diego, CA, USA) included multiple Welch *t*-tests, Mann—Whitney tests, Brown—Forsythe tests and Welch ANOVA tests, as well as Dunnett’s T3 post hoc test for multiple comparisons. Values are represented as means and standard deviations (SDs) or standard errors of the mean (SEMs), with a *p* value of 0.05 considered statistically significant (ns = *p* > 0.05; * = *p* ≤ 0.05; ** = *p* ≤ 0.01; *** = *p* ≤ 0.001; **** = *p* ≤ 0.0001). The performed tests and given significances are provided in the figure legends.

## 3. Results

### 3.1. Identification of SMKIs That Potently Inhibit In Vitro IAV Infections

We initially identified non-toxic SMKI concentrations (≥90% relative to DMSO-treated cells) using CellTiter-Glo (CTG), an ATP-based cell viability assay. Based on these data, we defined the 1× concentration ([1×]_max_) as the highest concentration with ≥90% relative viability (Figure 1A). Next, we infected A549 cells with either the pandemic A(H1N1)pdm09 A/Netherlands/602/09 (NL09) or the seasonal A(H3N2) A/Netherlands/241/11 (NL11) strain at a multiplicity of infection (MOI) of 1 in the presence or absence of selected kinase inhibitors at [1×, 0.5× and 0.25×]_max_ concentrations for up to 72 h post-infection (hpi). We observed dose-dependent viral titer reductions (from 2- to 1000-fold) using 14 of the 21 tested inhibitors (Figure S1). We focused our studies on seven inhibitors that showed >1-log_10_ (10-fold) titer reductions. As shown in Figure 1B and Figure S2, afatinib (AF), tucatinib (TU), neratinib (NE), avapritinib (AV), dabrafenib (DA), regorafenib (RG) and larotrectinib (LA) treatments all resulted in a reduction in viral titers for both NL09 and NL11 strains with variable potency and duration. AF (EGFR, HER2 and ErbB4 inhibitor) demonstrated the most potent and persistent level of reduction (>100- to 3000-fold). DA (c-/B-Raf), RG (VEGFR1/2/3, c-/B-Raf and PDGFRβ inhibitor) and NE (EGFR, HER2, VEGFR2 and Src inhibitor) also significantly reduced viral titers (DA ~10- to 100-fold; RG ~10- to 1000-fold; NE ~40- to 50-fold). 

While the effect of AF, DA and RG was significantly stronger in NL11-infected cells, NE as well as AV (PDGFRα inhibitor), LA (pan TRK inhibitor) and TU (HER2 inhibitor) treatment reduced the titers of both viruses (~10- to 30-fold) at the highest concentration [1×]_max_. All seven inhibitors reduced viral titers by ~1-log_10_ (10-fold) in both NL09- and NL11-infected cells; for LA, the NL09 reduction was only ~9-fold.

### 3.2. Effects of SMKI Treatment on In Vitro Cell Viability, Infectivity and Viral Spread

We next compared infectivity, cell viability and viral spread using quantitative immunofluorescence microscopy. In A549 cells infected with either NL09 or NL11 (MOI = 1) +/− the SMKIs at [0.5×]_max_ concentrations, we observed comparable reductions in relative infectivity in cells when treated with AF (NL09 = 73% vs. NL11 = 65%), AV (NL09 = 80% vs. NL11 = 81%) or RG (NL09 = 75% vs. NL11 = 80%). In contrast, the NL09 infectivity was more inhibited than the NL11 infectivity of A549 cells treated with LA (NL09 = 60% vs. NL11 = 91%), NE (NL09 = 70% vs. NL11 = 92%) or TU (NL09 = 86% vs. NL11 = 100%). Interestingly, although DA treatment decreased titers, the infectivity of A549 cells by either strain actually increased by comparable levels (NL09 = 117% vs. NL11 = 126%) (Figure 1C and Figure S2). The decrease in infectivity at 48 hpi could not be attributed to cytotoxicity. With the exception of AF, which only marginally decreased the relative viability, all SMKIs significantly increased the relative cell viability over the respective mock-infected treated controls (Figure 1D). To ensure our SMKIs did not inhibit virus replication through direct interactions, we preincubated the viral stocks with SMKIs and infected A549 cells with a 1:1000 dilution of pretreated virus (~MOI = 0.1). Pretreatment of the virus with RTKIs had no impact on viral titers, indicating that the observed effects on virus replication resulted from RTKI-induced perturbations of the host signaling (Figure 1E).

**Figure 2:**
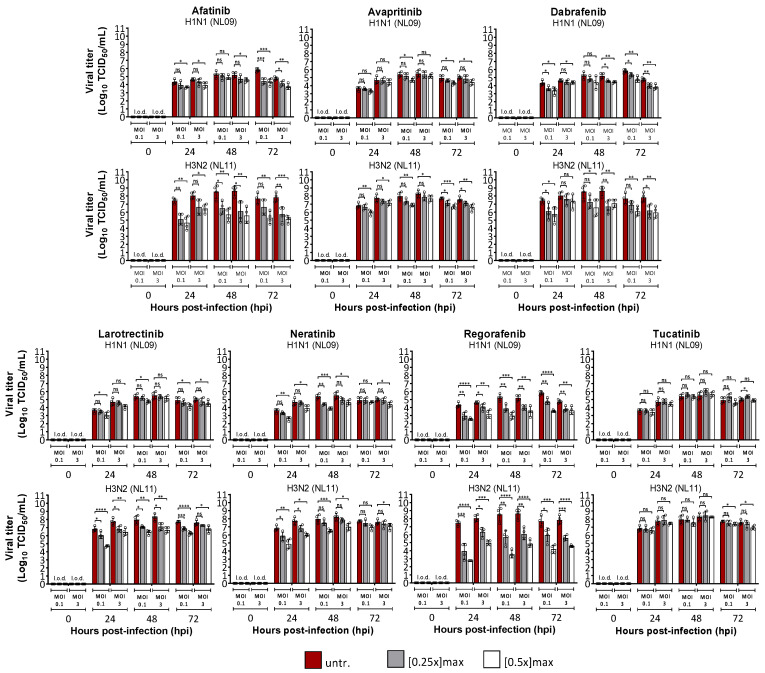
MOI-independent effects of SMKIs on IAV infection in vitro. A549 cells were infected with NL09 and NL11 at MOI = 0.1 (low) or MOI = 3 (high) for up to 72 hpi in the presence of SMKIs at [0.25×]_max_ (gray) and [0.5×]_max_ (white) concentrations or left untreated (red). At 24, 48 and 72 hpi, supernatants were collected, and viral titers were quantified by a TCID_50_/mL assay (*n* = 4). Means ± SDs are shown. l.o.d.: limit of detection. *, *p* < 0.05; **, *p* < 0.01; ***, *p* < 0.001; ****, *p* < 0.0001; ns, not significant. *p*-values were determined by Welch *t*-tests.

Next, we determined the effect of SMKIs on viral spread by comparing multicycle infection (MOI = 0.1) vs. single-cycle infection (MOI = 3) in A549 cells +/− the indicated SMKIs at [0.25× and 0.5×]_max_ for up to 72 hpi. Although SMKI treatment reduced viral titers by at least 10-fold at both MOIs, cells infected at MOI = 0.1 showed the highest reduction (AF 1000-fold; LA 100-fold; NE 100-fold; DA 100-fold; RG 10,000-fold; AV 10-fold; TU 10-fold) (Figure 2). Moreover, AF, LA, NE and RG treatments had a greater effect on virus replication at the early time points (24 hpi), particularly in cells infected with NL11, which has faster replication kinetics than NL09 (Figure 2), suggesting an effect on virus spread.

### 3.3. SMKIs Differentially Inhibit Various Steps of IAV Infection Cycle

**Figure 3:**
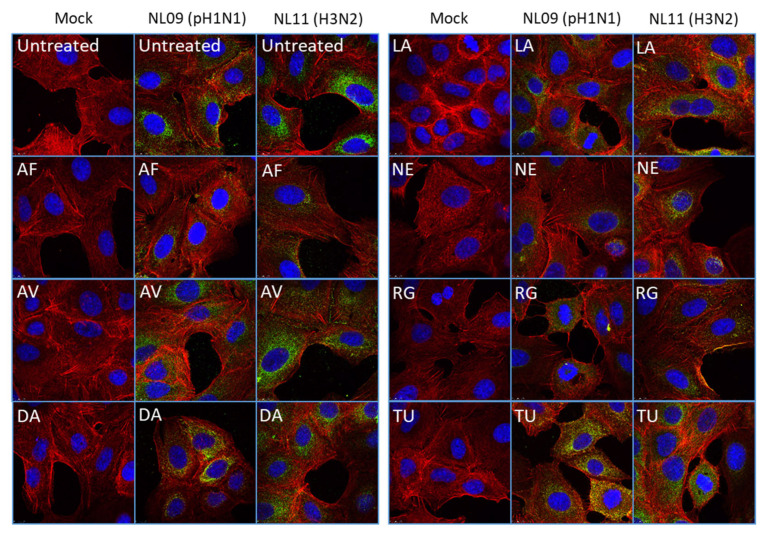
SMKI-specific effects on viral entry. A549 cells were pretreated with SMKIs for 2 h and then infected with either the NL09 or NL11 strain (MOI = 10) for 30 min +/− SMKIs [1×]_max_. Cells were fixed and permeabilized, and then virions were detected by anti-NP (green) antibody, F-Actin was detected by ActinRed-555 (red) and nuclei were detected using NucBlueLive ReadyProbes (blue). Virion localization was visualized by confocal microscopy (representative images from two independent experiments).

We aimed to identify which steps of the viral infection cycle were affected by each SMKI. To assess SMKI activity on viral entry, pretreated A549 cells were infected at a high MOI (MOI = 10) on ice to synchronize infection. At 0.5 hpi, we fixed and stained the cells and compared viral entry in the presence or absence of each SMKI candidate. Viral entry was inhibited by AF, DA, NE and RG treatments as detected by NP staining (Figure 3). However, no obvious effects on viral entry were observed in the AV, LA and TU treatments. 

Next, we determined the effect of SMKI treatment on viral RNA replication using the pPOLI-358-FFLuc luciferase-based reporter [29,30,31]. pPOLI-358-FFLuc and a transfection control plasmid (pmaxGFP) were co-transfected into A549 cells, and at 24 h post-transfection (hpt), cells were infected with NL09 or NL11 (MOI = 1) +/− the indicated SMKIs at [1× or 0.5×]_max_. Luciferase activity was determined at 48 hpt (24 hpi), normalized to GFP expression and reported as a percentage relative to untreated infected cells. 

**Figure 4:**
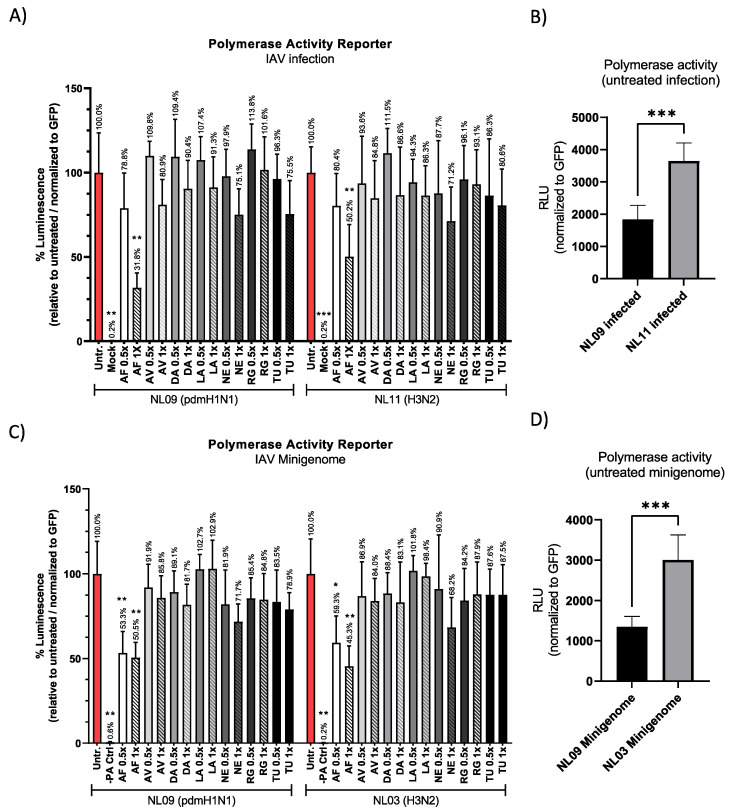
SMKIs affect IAV RNA replication. (**A**) A549 cells were transfected with pPOLI-358-FFluc and pmaxGFP plasmids. At 24 hpt, cells were infected with NL09 or NL11 at MOI = 1 +/− the indicated SMKIs at [0.5× or 1×]_max_ concentrations. At 48 hpt (24 hpi), luciferase activity was measured and normalized to GFP expression (MFI). (**B**) GFP-normalized polymerase activity of untreated NL09- or NL11-infected cells is shown. (**C**) A549 cells were transfected with pPOLI-358-FFluc and pmaxGFP plasmids and co-transfected with NL09 or NL03 minigenome plasmids. At 6 hpt, SMKIs were added to the medium. At 30 hpt (24 h of treatment), luciferase activity was measured and normalized to the GFP MFI. Bars indicate values relative to untreated cells normalized to GFP. (**D**) GFP-normalized polymerase activity of untreated NL09 or NL03 minigenome-transfected cells is shown. Triplicate measurements from triplicate samples (*n* = 3); error bars indicate ± standard deviation (SD). *, *p* < 0.05; **, *p* < 0.01; ***, *p* < 0.001. *p*-values were determined by Brown–Forsythe and Welch ANOVA tests compared to untreated cells.

Surprisingly, AF treatment had a significant effect on reporter activity (NL09 = 32% vs. NL11 = 50% at [1×]_max_); reporter activity was similarly reduced in both strains at the [0.5×]_max_ AF concentration (Figure 4A). In untreated NL11-infected cells, reporter activity was 2-fold higher than that in untreated NL09-infected cells, which correlates with the previously reported faster replication kinetics of NL11 compared to NL09 (Figure 4B). 

Next, we focused on viral polymerase activity and excluded effects on either entry or virion assembly/egress by using a minigenome reporter system. The pPOLI-358-Ffluc and pmaxGFP plasmids were co-transfected into A549 cells along with plasmids encoding NP and the polymerases PA, PB1 and PB2 of NL09 or the H3N2 strain A/Netherlands/213/03 (NL03), and at 6 hpt, SMKIs were added at either the [1×]_max_ or [0.5×]_max_ concentration. We used NL03 minigenome plasmids as we did not have access to those of NL11. The difference between NL09 and NL03 polymerase activity was ~2-fold (similar to NL09- vs. NL11-infected cells) (Figure 4D). Although a direct comparison between NL03 and NL11 has not been reported in A549 cells, virus growth kinetics previously reported in MDCK for NL03 were also ~2-log_10_ higher than those of NL09 [33], suggesting similar replication between the NL11 and NL03 H3N2 strains. At 30 hpt (24 h after treatment), we measured relative luciferase activity. Similar to our data in Figure 4A, AF at either the [0.5×]_max_ or [1×]_max_ concentration was the only SMKI to significantly reduce polymerase activity (Figure 4C). These data suggest that only AF inhibits viral polymerase activity in either infected or transfected cells.

**Figure 5:**
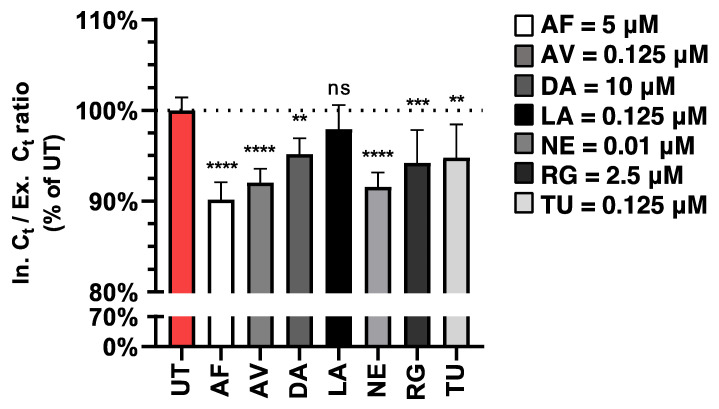
Treatments with different SMKIs limit IAV egress. A549 cells were infected at MOI = 5 in the presence of SMKIs [1×]_max_ for 24 h, at which point viral RNA in the supernatant and cells was quantified by qPCR. All values were normalized to an internal control and GAPDH. The ratios of intracellular (In) Ct to extracellular (Ex) Ct values were calculated and are shown as the percentage of untreated infected cells. All measurements were taken from three independent experiments (*n* = 7). Error bars indicate ± standard deviation (SD). **, *p* < 0.01; ***, *p* < 0.001; ****, *p* < 0.0001; ns, not significant (*p* > 0.05). *p*-values were determined by Welch *t*-tests compared to untreated cells.

To dissect the effects of our SMKI candidates on virion assembly/egress, we synchronized infections of A549 cells with NL09 (MOI = 5) on ice. Next, we excluded TPCK-trypsin from our infection media to limit subsequent rounds of entry and ensure “single-cycle” infections. We collected supernatants at 24 hpi, the earliest time point at which we detected robust viral accumulation (Figure 1B, Figure 2 and Figure S1), and cells were washed to remove traces of cell-associated extracellular/released virions. Viral RNA was extracted from both the supernatant (extracellular) and infected cells (intracellular) and detected by HA-specific qPCR. The ratios of intracellular to extracellular (I/E) viral RNA Ct values were calculated using HA Ct values that were normalized to GAPDH and CDV RNA internal controls and are represented as the percentage of untreated infected cells. This approach allows us to detect changes in extracellular viral RNA (egress) independently of any SMKI effects on viral entry or replication. All inhibitors except LA significantly reduced this relative I/E Ct ratio (Figure 5), suggesting that these inhibitors reduced viral egress, potentially contributing to the observed reduction in viral titers. 

### 3.4. Select SMKIs Exhibit Potent Antiviral Activity in IAV-Infected hPCLSs

**Figure 6:**
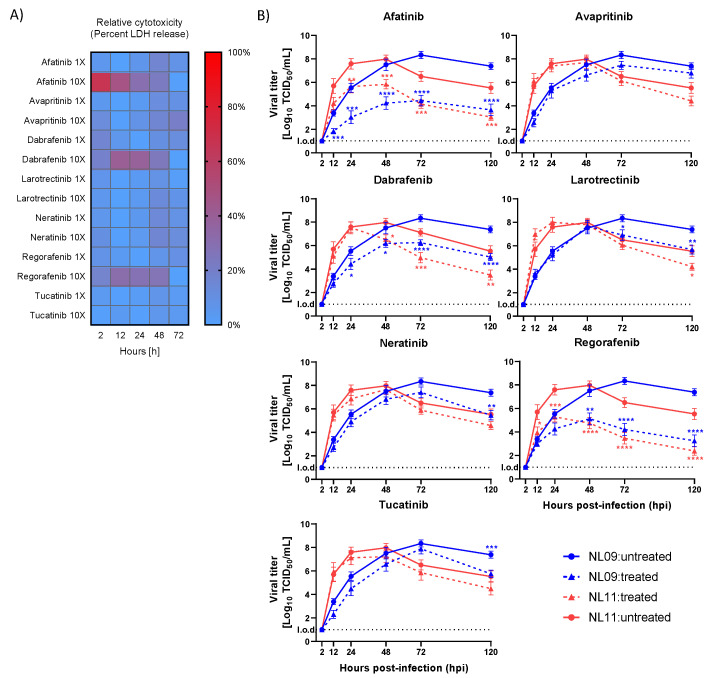
SMKIs impair ex vivo IAV infection. (**A**) Heatmap visualization of SMKI cytotoxicity in hPCLSs treated with [1×]_max_ and [10×]_max_ concentrations for up to 144hpi. At each time point, LDH release was measured using LDH-Glo Cell Viability Assay, normalized to the DMSO solvent control and calculated relative to 1% Triton-X-100-treated cells (positive control) (8 donors/*n* = 24). (**B**) hPCLSs were infected with NL09 or NL11 (10^5^ TCID_50_/200 uL) and incubated for 120 h with SMKIs: afatinib 5 uM (1×); tucatinib 1.25 uM (10×); neratinib 0.1 uM (10×); avapritinib 1.25 uM (10×); dabrafenib 10 uM (1×); regorafenib 2.5 uM (1×); larotrectinib 1.25 uM (10×). The virus was quantified by a TCID_50_/_mL_ assay (3 donors; *n* = 6/virus/condition/donor from 2 independent experiments); means are ± SEM. l.o.d.: limit of detection. *, *p* < 0.05; **, *p* < 0.01; ***, *p* < 0.001; ****, *p* < 0.0001. *p*-values were determined by Mann–Whitney tests compared to untreated cells.

First, we identified the highest tolerable SMKI concentrations using [1×]_max_ derived from the A549 titrations above. We measured inhibitor tolerability using human precision-cut lung slices (hPCLSs) as an ex vivo model from eight donors (*n* = 24) at either [1×]_max_ or [10×]_max_ with the LDH-Glo bioluminescent cytotoxicity assay to detect LDH release, a marker of cytotoxicity, with an established 20% cytotoxicity cut-off [22,34]. Measurements were normalized to the positive control treatment (0.1% Triton-X 100); DMSO-treated hPCLSs served as negative controls (Figure 6A). Although none of the seven tested inhibitors exceeded our cut-off at [1×]_max_, AF (50 μM), DA (100 μM) and RG (25 μM) increased cytotoxicity above the 20% cut-off at [10×]_max_; therefore, SMKIs were only used at [1×]_max._

We previously established an infectious dose of 10^5^ TCID_50_ and showed that both the NL09 and NL11 strains efficiently infected various cell types, including type I/II pneumocytes [22]. Using the same dose, we infected hPCLSs from three donors (*n* = 6/virus/condition) with either NL09 or NL11 and then treated them with SMKIs (AF 5 μM; AV 1.25 μM; LA 1.25 μM; NE 0.1 μM; DA 10 μM; RG 2.5 μM; TU 1.25 μM) and assessed viral titers at 2, 12, 24, 48, 72 and 120 hpi. The treatments reduced viral titers by ~10-fold (AV treatment) to more than 1000-fold (AF and RG treatments) (Figure 6B). We observed early inhibition of virus replication within 12 hpi in AF-, AV-, DA-, NE-, RG- and TU-treated samples, which was sustained for 120 hpi. In contrast, no appreciable effect was observed in LA-treated cells before 72 hpi. This observed reduction was consistently significant after 48 hpi in AF-, DA- and RG-treated infected hPCLSs, validating these SMKIs as potent antivirals in a biologically relevant human ex vivo model.

### 3.5. Tested SMKIs Have a High Genetic Barrier of Resistance

**Figure 7:**
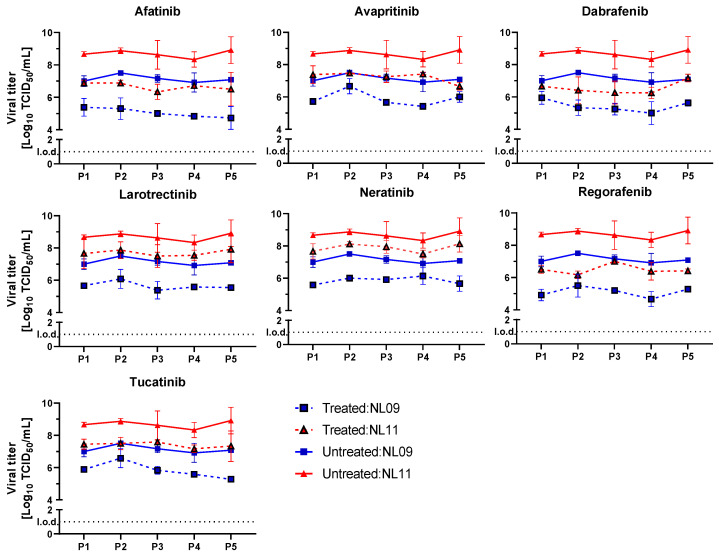
SMKI inhibition of IAV is maintained over serial passaging. The stability of SMKI treatment on NL09 and NL11 replication was determined by five serial passages on MDCK cells infected at MOI = 0.001 for 72 h in the presence of the [1×]_max_ SMKI concentrations (*n* = 4) at each passage. At each passage, viral titers were quantified to inoculate the next passage at the same MOI; means ± SDs are shown. l.o.d.: limit of detection.

We serially passaged NL09 and NL11 viral stocks in the presence or absence of our selected SMKIs to assess their genetic barrier of resistance. We used MDCK cells as they are extremely permissive and provide an advantageous environment for IAV growth. MDCKs were infected at MOI = 0.001 for five serial passages in the presence of SMKIs at [1×]_max_ concentrations. We mitigated strain-dependent differences in peak titers by measuring viral titers after each passage and infected the next passage again at MOI = 0.001. As controls, we also passaged the same viruses in the absence of SMKIs. The level of viral titer inhibition relative to controls (DMSO) was consistent throughout the multiple passaging and comparable to what we observed in A549 cells (Figure 7). Although we could not rule out the rise of any mutations during virus passaging in the presence of our inhibitors, our data suggest that no mutations conferring resistance accumulated in the viruses passaged in the presence of SMKIs. 

## 4. Discussion

We recently identified six potent antivirals of IAV from a screen of FDA-approved NRTK inhibitors (NRTKIs) [22]. Building on our recent findings, we screened 21 additional FDA-approved SMKIs that included RTK and serine/threonine kinase inhibitors. We identified seven promising candidates that potently inhibited in vitro IAV infections in A549 cells and reduced viral titers by 10- to 3000-fold. We identified differential inhibition of various steps of the viral infection cycle by these SMKIs. Importantly, the antiviral activity of these inhibitors was validated in a biologically relevant ex vivo system using hPCLSs from 11 donors in total. Based on the observed increase in relative cell viability during SMKI treatment, impaired infectivity (replication and spread), not cytotoxicity, is most likely the major driver of the robust inhibition of IAV infection we observed in vitro and ex vivo.

RTKs respond to various stimuli and relay “outside-in” signals to regulate multiple cellular processes via distinct pathways that often overlap through shared signaling nodes including FAK, Grb2/SOS, PI3K/Akt and Raf/MEK/ERK. These shared signaling nodes are differentially and temporally phosphorylated to mediate anti- and/or pro-apoptotic responses that must be balanced to ensure cellular viability [35,36]. Indeed, viruses including IAVs have evolved to exploit kinases regulating these signaling pathways through direct phosphorylation of either viral proteins or host proteins that are essential for efficient replication [15,16,17,37,38,39,40,41,42,43,44,45,46,47,48,49,50,51]. Most SMKIs we tested target RTKs such as EGFR, VEGFR, PDGFR and TrkA, all of which have been reported to regulate multiple steps of the IAV infection cycle, including viral entry, RNA replication and virion assembly/egress [39,40,41]. EGFR regulates viral entry through PI3K/AKT, Raf/MEK/ERK and Src signaling that promote IAV uptake [52]. Moreover, through intricate signaling cascades that recruit the Grb2/SOS complex, PI3K/Akt, PKC and Raf/MEK/ERK, IAV-induced EGFR activation facilitates RNA replication and host immune responses [18,35,53,54]. 

It is not surprising that treatment of infected cells with our most potent SMKI (AF), which inhibits EGFR family kinases (EGFR, HER2 and ErbB4), impaired viral entry, polymerase activity and viral egress. A previous study showed that AF treatment of A549 cells infected with IAV laboratory strains (PR8 and WSN) and pandemic H1N1 (CA09) showed only a modest (~2- to 5-fold) effect on viral replication [55]; nevertheless, they were able to show that AF’s major target, EGFR, played a critical role in IAV replication. It should be noted that in that study, the authors used a much lower dose of AF than our study (1μM vs. 5μM) and looked at an earlier time point (15 hpi), which may explain the difference in the antiviral potency of AF they observed. Although to a lesser extent than AF, a robust reduction in viral entry was also observed in DA-, NE- and RG-treated cells. AF likely limits viral entry through EGFR inhibition and possibly through ErbB4. This is consistent with EGFR’s established role in viral entry and the fact that neither NE nor TU inhibits ErbB4. AF was more effective at limiting viral entry than NE (inhibits EGFR and HER2). Moreover, because TU treatment (only targets HER2) had no effect on viral entry, it is likely that HER2 is dispensable for this process. DA selectively inhibits B/c-Raf with similar selectivity, and RG is ~10-fold more selective for c-Raf than B-Raf [56,57]. Therefore, the clear inhibition of viral entry by DA and RG treatment we observed is consistent with the well-established role of Raf/MEK/ERK signaling in viral entry [45,58]. Interestingly, Lesch et al. reported that RG treatment reduced IAV replication in vitro by impairing endosomal acidification and membrane fusion [58]. Surprisingly, that study found that RG treatment was less potent in primary bronchial cells than in A549 cells. This contrasts with our results, which show that the reduction in hPCLSs was much more potent than the viral reduction in A549 cells (~4 log_10_ vs. ~2 log_10_ reduction). The cause for this discrepancy might be due to differences between well-differentiated epithelial cells and hPCLSs. Furthermore, that study used a single donor (triplicate from a single experiment), whereas we used at least three donors (*n* = 6 from two independent experiments) to limit donor bias. Nevertheless, both studies point to RG-mediated inhibition of the Raf/MEK/ERK pathway, which is crucial for endosomal acidification via V-ATPase activity [45]. 

Interestingly, with the exception of AF, none of our SMKIs that target EGFR, HER2, PDGFR or B-/c-Raf significantly impaired viral polymerase activity. Given that none of them targeted ErbB4, our data suggest that ErbB4 inhibition is the main driver of AF-mediated polymerase activity impairment. To our knowledge, a direct link between ErbB4 (not EGFR) and IAV polymerase activity has not previously been described, and a mechanism for this is yet to be defined. A recent study suggested that B-Raf is dispensable for viral entry and used the B-Raf inhibitor vemurafenib [59]. However, both DA and RG have a higher selectivity for c-Raf (10-fold) and B-Raf (10- to 100-fold) than vemurafenib and may have been more efficient at B-Raf inhibition; therefore, we cannot confirm or contest the results of that study. It is tempting to speculate that c-Raf, not B-Raf, is the major driver of EGFR-mediated Raf/MEK/ERK signaling during IAV infections and affecting either entry, replication or both.

EGFR-mediated Raf/MEK/ERK activation is also required for nuclear export of the viral ribonucleoprotein (vRNP) and subsequent virion budding via regulation of phosphorylation-dependent Crm1 nuclear/cytoplasmic shuttling [18]. Inhibition of Raf/MEK/ERK activation during IAV infection results in vRNP nuclear retention and a subsequent reduction in viral titers [17]. Accordingly, all tested SMKIs, except LA, caused a reduction in the intracellular-to-extracellular (I/E) vRNA ratio, suggesting impairment of viral egress by a mechanism that remains to be elucidated.

PDGFRs, such as EGFR, are key players in chronic tissue remodeling in asthma, bronchitis and pulmonary fibrosis [60,61]. Recent studies have implicated PDGFRβ and GM3 gangliosides in viral entry through a Raf/MEK/ERK-dependent but PI3K/Akt-independent mechanism [62]. Moreover, we and others have shown that SMKIs that inhibit PDGFRs (PDGFRα or PDGFRβ) significantly inhibit IAV polymerase activity and RNA replication [18,22]. TrkA has been implicated in regulating multiple steps of IAV replication using inhibitors and RNA silencing [18]. However, the inhibitors used in that study also target PDGFRα and PDGFRβ, both of which have been shown to regulate IAV entry and replication. Indeed, we saw a significant reduction in viral titers in both in vitro and ex vivo IAV infections using the pan-Trk (inhibits TrkA, TrkB and TrkC) inhibitor LA. However, we did not observe significant differences in any single step of viral replication we tested. A possible explanation for the significant reduction in viral titers we observed in the LA treatment is that it is the result of small cumulative effects on multiple steps of IAV infection previously reported by Kumar et al. [18]. It should be noted that RNA silencing of TrkA would affect TrkA/HER2 signaling and TrkA kinase-independent interactions with binding partners (e.g., CD44, FAK, Actin, Arp2/3 or RhoA), which may have contributed to the antiviral effect they observed. 

VEGFRs (VEGFR1, VEGFR2, VEGFR3) are expressed on endothelial cells, monocytes, neuronal tissues and mesenchymal cells, where they regulate angiogenesis, survival and proliferation via FAK, PI3K/Akt or Raf/MEK/ERK signaling [63,64]. Moreover, VEGFR signaling was recently implicated in the cytokine storm response of severely ill IAV patients as well as in IAV-infected pregnant mice, suggesting a role in IAV pathogenesis via yet-to-be-determined molecular mechanisms [65,66]. Inhibition of different members of the VEGFR family has been shown to impair IAV replication [58]. NE and RG inhibit the kinase activity of VEGFRs; however, the contribution of VEGFR inhibition to the potency of these two SMKIs in our study is not readily clear. VEGF/VEGFR expression and signaling are dysregulated in the A549 lung adenocarcinoma cell line [67]. In contrast, hPCLSs maintain the 3D tissue architecture and cellular composition of the lungs, including endothelial cells, ATI/II cells and mesenchymal cells of the parenchyma that express physiological VEGF and VEGFRs levels [23,24,25,26]. This could explain the more potent antiviral effect of NE and RG we observed in hPCLSs compared to A549 cells.

In summary, we identified and validated seven FDA-approved SMKIs, already in clinical use for other diseases, as potent IAV antivirals. These data may guide the development of next-generation antivirals with fine-tuned selectivity for virus–host interactions and rationale for maximizing SMKI antiviral efficacy through combination therapies. A major disadvantage of DAAs is the rapid accumulation of escape mutations that give rise to drug-resistant viruses within a few passages in vitro; this has also been readily observed in the clinical setting [12,13,15,68,69]. We observed no evidence for the emergence of resistance variants during serial passaging of the viruses in the presence of the tested SMKIs, indicating that, unlike virus-directed antivirals, host-directed antivirals have a much higher barrier of resistance and are minimally susceptible to escape mutations. 

The established safety and bioavailability data of the tested SMKIs warrant further clinical evaluation of these compounds as potential influenza treatments. In the clinical setting, localized delivery of these SMKI to sites of IAV replication (respiratory tract) could increase tolerance and potentially broaden the range of therapeutic dosage. Although “antiviral efficacy” is typically based on the reduction in viral titers/loads, it is perhaps more important to also consider the effect on immune responses when investigating host-directed compounds. Indeed, the effect of SMKIs on resident or infiltrating immune cells should be considered to limit the potential of dysregulating the immune response. Importantly, the therapeutic window is likely to be different than that of virus-targeted antivirals (DAAs) and should be considered when establishing efficacy. Because many viruses rely on the same conserved host kinases for efficient replication, pathogenesis and transmission [52,54,65,70,71,72,73,74,75,76,77,78,79,80,81,82,83,84], our findings may have broader implications for the treatment of other viruses.

## Data Availability

Not applicable.

## Supplementary Materials

The following supporting information can be downloaded at: https://www.mdpi.com/article/10.3390/v14092058/s1, Figure S1: SMKI candidate screening against NL09 and NL11 infections. Figure S2: Effect of selected SMKI treatments on NL09 and NL11 infections; Figure S3: Immunofluorescent detection of SMKIs’ effects on cell viability and infectivity during infection.
