# Supplementary material for: FDA-Approved Inhibitors of RTK/Raf Signaling Potently Impair Multiple Steps of In Vitro and Ex Vivo Influenza A Virus Infections
Source: Viruses. 2022 Sep 16;14(9):2058. doi: 10.3390/v14092058 (PMC9504178; doi:10.3390/v14092058)
Supplement: Supplementary file 1 [file viruses-14-02058-s001.zip › viruses-1864454-supplementary.pdf]

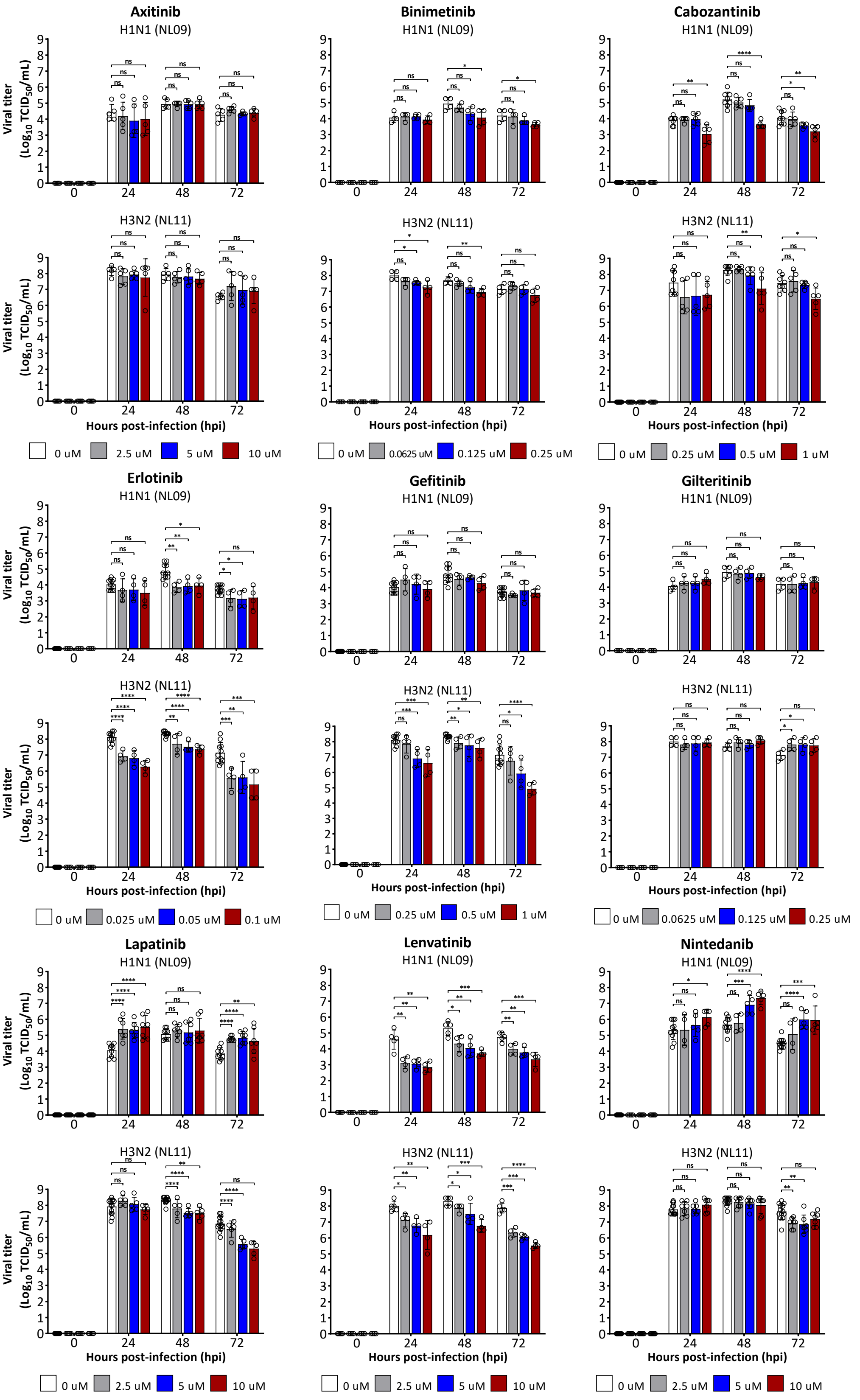

Axitinib
H1N1 (NL09)
H3N2 (NL11)
0 uM
2.5 uM
5 uM
10 uM
Binimetinib
H1N1 (NL09)
H3N2 (NL11)
0 uM
0.0625 uM
0.125 uM
0.25 uM
Cabozantinib
H1N1 (NL09)
H3N2 (NL11)
0 uM
0.25 uM
0.5 uM
1 uM
Erlotinib
H1N1 (NL09)
H3N2 (NL11)
0 uM
0.025 uM
0.05 uM
0.1 uM
Gefitinib
H1N1 (NL09)
H3N2 (NL11)
0 uM
0.25 uM
0.5 uM
1 uM
Gilteritinib
H1N1 (NL09)
H3N2 (NL11)
0 uM
0.0625 uM
0.125 uM
0.25 uM
Lapatinib
H1N1 (NL09)
H3N2 (NL11)
0 uM
2.5 uM
5 uM
10 uM
Lenvatinib
H1N1 (NL09)
H3N2 (NL11)
0 uM
2.5 uM
5 uM
10 uM
Nintedanib
H1N1 (NL09)
H3N2 (NL11)
0 uM
2.5 uM
5 uM
10 uM
Viral titer
(Log$_{10}$ TCID$_{50}$/mL)
Hours post-infection (hpi)
0
24
48
72

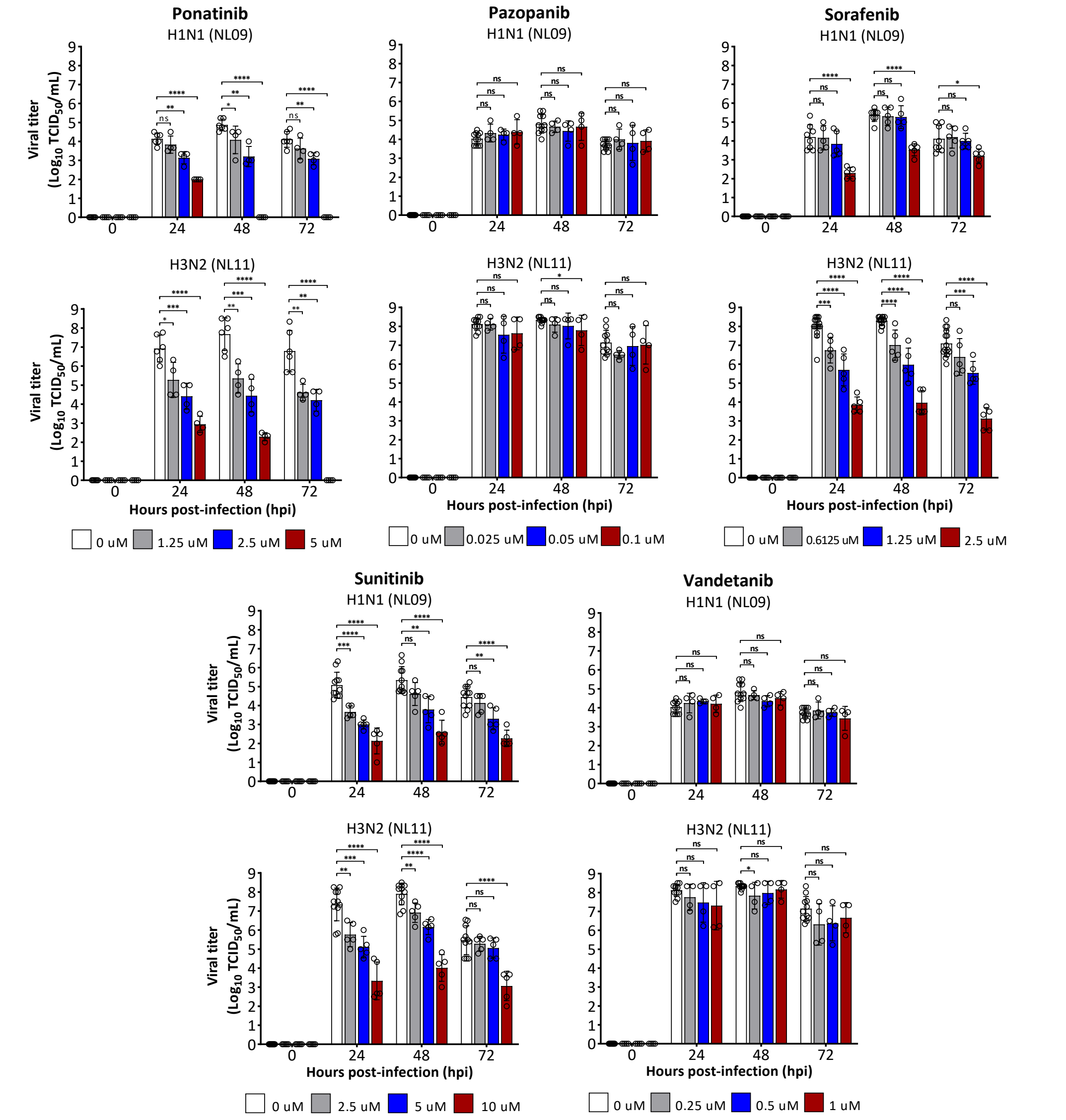

Supplemental Figure S1: SMKI candidate screening against NL09 and NL11 infections. A549 cells were infected with NL09 or NL11 at MOI=1 and incubated for 72h +/- indicated SMKIs at $[0.25x, 0.5x \text{ or } 1x]_{max}$ concentrations. At 24, 48, and 72 hpi, supernatants were collected, and viral titers quantified by $TCID_{50}$/ml assay (n = 4). Means ±SD are shown. *, P<0.05; **, P<0.01; ***, P<0.001; ****, P<0.0001; ns, not significant (P>0.05).

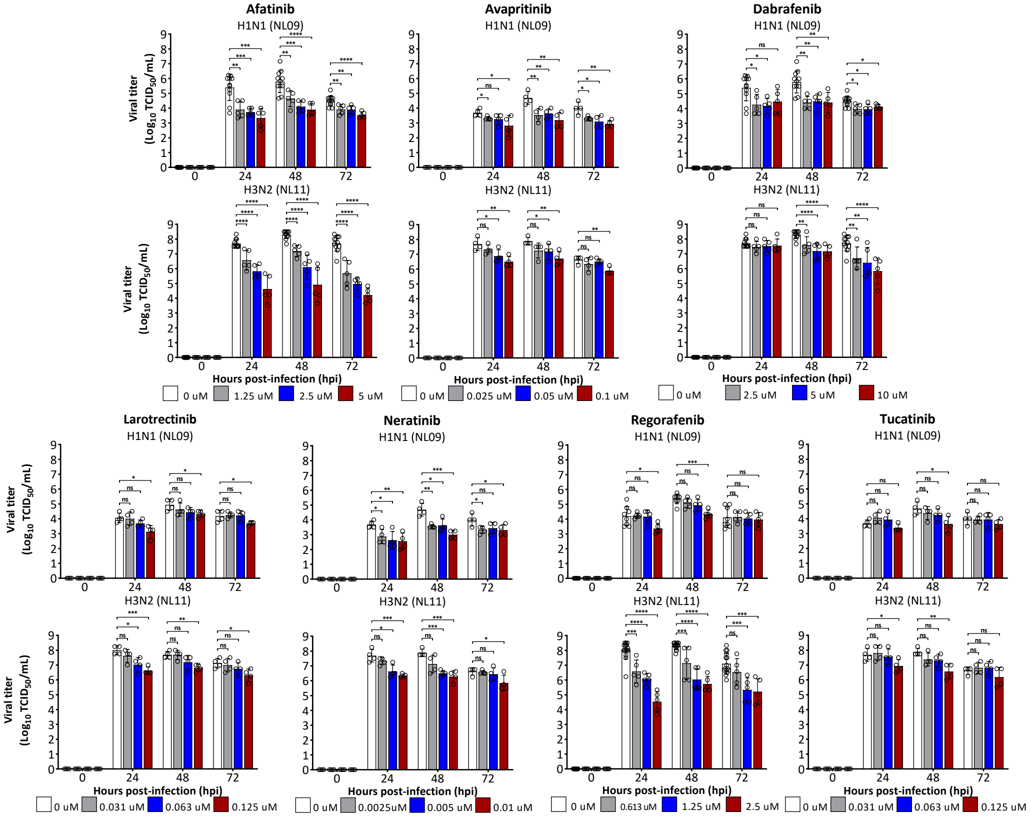

Supplemental Figure S2: Effect of Selected SMKI treatment on NL09 and NL11 infections. ***Related to Fig. 1B***. A549 cells were infected with NL09 or NL11 at MOI=1 and incubated for 72h +/- indicated SMKIs at $[0.25x, 0.5x \text{ or } 1x]_{max}$ concentrations. At 24, 48, and 72 hpi, supernatants were collected, and viral titers quantified by $TCID_{50}$/ml assay (n = 4). Means ±SD are shown. *, P<0.05; **, P<0.01; ***, P<0.001; ****, P<0.0001; ns, not significant (P>0.05).

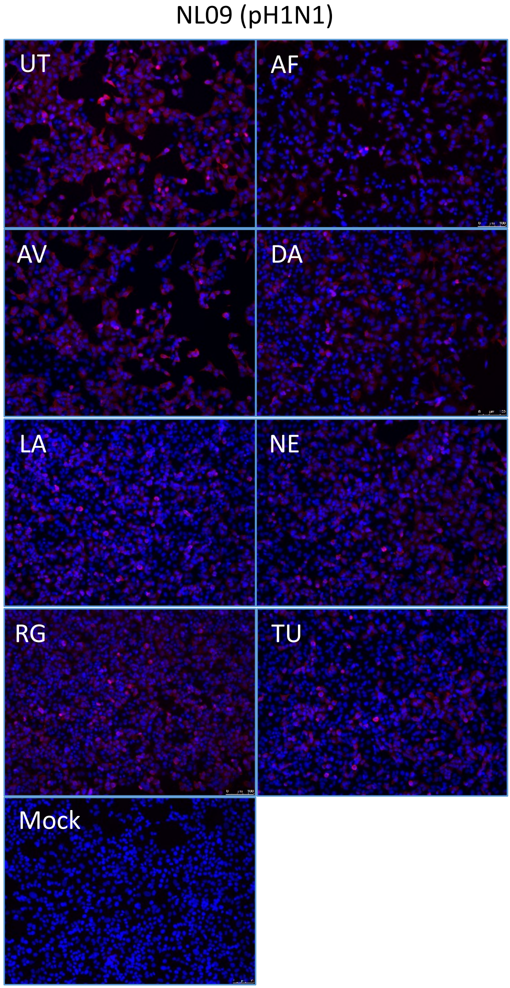

Supplemental Figure S3. Immunofluorescent detection of SMKIs' effects on cell viability and infectivity during infection. ***Related to Fig. 1C and 1D.*** A549 cells were infected with NL09 or NL11 at MOI=1 +/- indicated SMKIs at $[0.5x]_{max}$ concentration for 48h. Fluorescence microscopy pictures were captured using a Leica DMi8 fluorescence microscope (representative field shown from an n=4/condition). Virus- infected cells were detected by anti-IAV NP antibody (red), and nuclei were detected using NucBlue Live ReadyProbes (blue).
